# The Voluntariat: A Frieirean framework to understand the nature of undergraduate international (medical) experiences

**Published:** 2016-12-05

**Authors:** Seemi Qaiser, Helen Dimaras, Paul Hamel

**Affiliations:** 1 University of Toronto; 2 Department of Ophthalmology & Vision Sciences, Faculty of Medicine & Division of Clinical Public Health, Dalla Lana School of Public Health, University of Toronto; 3 Department of Laboratory Medicine & Pathobiology, Faculty of Medicine, University of Toronto

## Abstract

Despite literature documenting limited and asymmetrical benefits along with ethical issues, short-term international volunteering is increasingly popular among North American university students as a perceived advantage when applying to professional healthcare schools or the job market. Academic institutions are also encouraging students to pursue international experiences in order to cultivate values as global citizens. These experiences are most typically limited to economically privileged students. Furthermore, international activities in developing countries often lack a pedagogy of social justice and may confirm a simplistic understanding of development. Brazilian educator Paulo Freire’s “liberation pedagogy” provides a framework for understanding the limitations of international volunteering, whereby the presence of privileged volunteers implementing Western models of development may hinder aspects of local movements. Regardless, university students face intense competition in accessing opportunities, such as medical school, and pay large sums to participate in volunteering to strengthen their academic credentials. We propose that these students form “the voluntariat.” They simultaneously play two roles by, first, contributing to the conditions that oppress the very communities in which they volunteer and, second, by playing a role as objects of oppression by the liberal institutions of learning and employment to which they are attempting to gain access.

## Introduction

*She was doing HIV awareness… and she’s teaching people how to use condoms…you’re a seventeen-year-old girl straight out of high school in the UK and you’re going to teach adults [how to use a condom] …We actually just tolerate these things that you do ‘cause we like need your money and you have that colonial relationship…but nobody buys the bullshit.* (Kenyan activist Binyavanga Wainaina on meeting an international volunteer in Kenya)^1^

Having spent many late nights scouring online premedical forums in search of the elusive factor that guarantees admission to medical school, international volunteering seemed to be the hot ticket in town. Of those who responded to the question in the 2015 post-Medical Colleges Admission Test (MCAT) questionnaire (n = 26,459), 27% reported having volunteered internationally.^2^ This perceived advantage seems to continue in medical school. In 2012, 30.4% of graduating US medical students participated in short-term medical work, up 5.4% from the 25% in 2008 who participated in global health experiences.^3^,^4^ As a medical school-hopeful with extensive international experience, I (author SQ) find the potential effect of these numbers troubling because these short-term global health experiences may be creating a new generation of medical practitioners harbouring simplistic understandings of the systemic forces shaping the lives of their patients, who are increasingly influenced by globalization.

### University trend: encouraging international service and education

In my interviews for scholarships and jobs, the background experience that I believe gives my applications some advantage over others is my past research experience on eye cancer in Kenya and clean cook stove technology in India. Although I make an effort to realistically convey my limited contributions to these international projects and distinguish them from service work, I perceive the individual interviewing me for the coveted position is already focused on my global experience. This fascination with an otherwise 2-in-1 combination of tourism and altruism extends beyond admission committees and employers to higher-level academic institutions seeking to increase the employability of their graduates.

Despite the increasing number of students engaging in international experiences, emerging literature in global health and international development has become increasingly critical of international volunteering. International activities are typically short-term (1–8 weeks)^5^ where volunteers, rather than the communities, tend to be the main beneficiaries.^3^ Another critique is that volunteers may engage in ethically dubious situations in medical settings.^6^ Regardless, universities appear to be encouraging their students to participate in international experiences through studying and volunteering abroad.^7^,^8^ In an article suggestively titled, “Scrambling for Africa? Universities and global health,” Crane ponders, with reasonable skepticism, whether the increasing popularity of global health academic programmes is a race for globally-ranked universities to brand themselves in competition with other universities.^8^

Crane’s allusion to the 1884 Berlin Conference, where Africa was divided amongst European nations, is an eerie reminder of Africa’s colonial past and implies that this exploitive relationship may not be over.^8^ Canadian universities seem to be following a similar trend of scouting new territories to expand their influence. A recent discussion paper by University of Toronto’s President Gertler highlights “Strengthening Key Partnerships: Towards an International Strategy” as one of the university’s top three priorities including forming international partnerships, student mobility, international student recruitment, international presence, and interdivisional co-ordination.^9^ In this context, it is suggested that increasing student exposure to and understanding of other cultures via studying and living abroad will lead to the production of “global citizens.” With no formal definition of global citizenship provided, the assumption may be that international experiences provide opportunities for students to challenge their everyday thinking^10^ and to see themselves embedded in complex local and global systems through new cultural experiences. But maybe not.

### International experiences are expensive and inaccessible to most students

President Gertler’s report recognizes that participation in international experiences seems to be limited to select students – those who can afford the high costs and do not face cultural norms that may prevent them from engaging in international travel.^9^ However, the underlying factor in both cases may be lack of funds. Studying or volunteering abroad is expensive. Thus, it typically remains an activity only for financially-elite students who can bear the financial burden of paying for an international “experience” and forgoing the summer income typically used to offset pending tuition fees or debt. The tourism aspect becomes commodified into experiences for the consumption of middle class youth demarcating class lines. The accumulation of “cultural capital” is used as “an entry qualification” for particular lines of work (medicine, for instance), transforming it into economic capital.^11^ This exclusive access to international experiences may give these students an advantage when applying to medical school over other candidates who were unable to afford such a trip. A Canadian study found higher-income groups to be overrepresented in Canadian medical schools with 57.6% of medical students reporting parental household incomes of more than $100,000/year,^12^ corroborating the apparent bias for students in the same socioeconomic status who are able to “volunteer” abroad.

The disparities among those interacting in these situations can give rise to deleterious consequences. Tausch et al. have shown that positive contact of disadvantaged groups with advantaged groups can decrease motivation for collective action by leading to decreased in-group identification and positive attitudes towards the advantaged group.^13^ Interaction of community members with Western volunteers modelling Western lifestyles can foster the belief that social mobility is possible through individual efforts, leading to increased identification with the aspirational volunteers and diminished identification with other community members. Thus, the presence of volunteers can be detrimental to local movements by reducing their anger at the inequality^13^ without altering the conditions giving rise to the inequality. On the volunteers’ side, it is also unclear as to how the financially privileged medical volunteers translate this learning to benefit the communities since it is physicians from minority (including low-income groups) backgrounds who are more likely to work in underserved areas and with minority patients.^12^ Minority patients, too, are more satisfied when treated by a minority physician.^12^

### International experiences can result in a simplistic understanding of development

What are future physicians learning from these experiences? International work can widen perspective on different ways of living around the world and expose young doctors to new pathologies.^14^,^15^ However, it has also been suggested that it fails to provoke critical thinking regarding the basic health inequities and lacks the social justice pedagogy critical for understanding the political and economic factors responsible for these gradients in global health.^10^,^15^,^16^ A social justice framework encourages students to examine the root causes of inequalities and challenge narratives that blame individuals alone for their circumstances. Indeed, as Arundhati Roy^18^ has stated in the context of non-governmental organizations (NGOs):

In order to make sure their funding is not jeopardized and that the governments of the countries they work in will allow them to function, NGOs have to present their work in shallow framework, more or less shorn of a political or historical context.

Many international volunteer programs are not designed to imbue development principles and rely on the sensationalist imagery of thankful smiles in the midst of extreme poverty to provide a cultural experience.^10^ With no frameworks to orient their understanding, volunteers resort to “lotto logic” to explain root causes like poverty, where individuals are assumed to be born poor simply because of bad luck.^16^ This creates a flawed narrative of development in which the enthusiasm and good intentions of youth are enough to combat the fateful consequences of poverty, ill health, and poor education. The qualifications, competencies, or ethics surrounding this youthful involvement remains an afterthought.^16^

### Lack of anchoring frameworks prevents the development of global citizens

Volunteers typically perceive themselves as belonging to a separate category from those they encounter in foreign, usually, poorer countries. This separation allows multiple dichotomies to flourish: “us/them,” “local/global,” “developed/developing.” 17 In one study, volunteers returning home to the United Kingdom were found to have struggled to translate their learning from the global scale to local settings. In these cases, the issues felt distant and volunteers felt unsure regarding their next actions.^10^ This is a possible result of i) a myopic understanding of the stark phenomena that volunteers may witness during their transitory “volunteer encounter” and ii) the expectation that the relationships that were forged will not persist.^17^ It is naïve to think that cross-cultural understanding will grow spontaneously, solely by occupying and interacting with less-privileged people of the developing world.^19^ Images of smiling western volunteers with members of the local communities, often used for marketing of study/volunteer-abroad programs, reproduce the reductionist kumbaya myth^20^ of “we are all one race so why can’t we just get along?” This idea serves to erase boundaries of race, class, and other hierarchical forces that distinguish the volunteers from the community members and homogenizes the two groups into members of the human race who can eliminate all inequalities if only they hold hands and sing.

### Using Freire’s “Liberation Pedagogy” to contextualize international volunteering

Brazilian educator Paulo Freire’s “liberation pedagogy”^21^ provides one framework to understand the dynamics between the volunteers and the communities they are working with; it has been used to discuss voluntourism before.^10^,^16^ Freire formulated a dichotomy that designated categories of “oppressor” and “oppressed.” As part of his thinking, he further outlined the mechanisms used by the former to maintain oppression. Freire advocated for change led *by* the oppressed rather than *for* them, criticizing teachers who unknowingly perpetuate domination when working with the oppressed.^22^ Similarly, the agenda for international volunteer programs often does not reflect the needs of the community and imposes development premised on Western economics, led by volunteers who adhere to and reproduce Western lifestyles and values.^16^ Western theories of development are used in these projects. These promote the integration of marginalized communities into the global system of capitalism, the seemingly “good, organized, and just” system into which they have yet to be integrated.^21^ Volunteers promote “the bootstrap myth”, the idea that anyone, regardless of circumstances at birth, can overcome all barriers and acquire the American Dream if they simply work hard enough. Volunteerled projects perpetuate this false notion in the communities in which they operate, suggesting to community members that they too can achieve social mobility if only they participate in the volunteers’ programming. Positive interactions by the community members with the volunteers can lead to identifying with them, thereby promoting an individualistic mentality and preventing community members from perceiving themselves as part of a collective, oppressed class.^13^

### ‘Voluntariat’: buying the right to work through international volunteering

I propose a new term to account for the particular position that international volunteers occupy in relation to development and economic conditions in the 21^st^ century: “voluntariat.” Where the “proletariat” forms a class of workers who do not own capital and must sell their labour, the voluntariat is its counterpart. The voluntariat is driven by i) “the white man’s burden” – the responsibility that the Western world feels to improve the conditions of the developing world and ii) the need to gain an advantage in an employment market where higher education admission to professional schools is increasingly competitive, despite higher education not guaranteeing a liveable income in his/her field of education. Produced by the intersection of globalization and neoliberalism, the voluntariat constitutes a pool of young, unskilled, middle-class labourers from the global North, available internationally for short terms for free or, more typically, by paying a fee. In a global capitalist economy, their existence seems fantastical. And yet, the exploitation of the worker has been so intensified that individuals are willing to pay to gain access to the employment market. In traditional volunteering, the volunteer does not receive monetary compensation. In its current manifestation, the voluntariat furnishes her/his own reproduction of labour by paying for the experience. Simultaneously, while attempting to provide goods and services for the maintenance of the labour force in the community in which s/he serves, the voluntariat promotes the integration of these communities into the capitalist system by creating health workers who become objects for exploitation, opening new niche markets in the process. In this way, the voluntariat not only contributes to the oppression of the community in which they operate, but is simultaneously the object of oppression by liberal institutions, in this case the employment market and graduate schools.

### International experiences need not be completely dismissed

The U of T’s President’s Report suggests that international experiences for university students could accompany the quest for recruiting graduate students from “key markets” abroad.^9^ This could raise the profile of the university and form partnerships with universities of “comparable quality”, while contributing to the development of other institutions in the vaguely dubbed “specific parts of the world, such as Africa.”^9^ With university presidents under high pressure to fundraise,^22^ international expansion provides an avenue to enter new markets, opening up new opportunities for profits. The discourse on development and mobility is rife with undercurrents of elitism, power, and, though sounding quite cliché, money.

In order to navigate the forces that shape the lives of patients, internationally-based education for medical students must demonstrate awareness of these factors to form physicians who are also active global citizens. The aim of this paper is not to discourage all international experiences but to understand that they must be accompanied by a pedagogy based on social justice. Indeed, my own undergraduate experience benefitted greatly from the international research work I was able to pursue by allowing me to apply theories from the classroom, work more intimately with my professors, and interact with professionals from a variety of backgrounds. Volunteers must be encouraged and trained to identify the ways they, themselves, might be complicit in the suffering of the populations with which they are working rather than seeing themselves as saviours. Instead of relying on sensational imagery, medical education must prioritize reflection on the systematic causes of health inequities using a social justice lens.
